# Hereditary angioedema: Assessing the hypothesis for underlying autonomic dysfunction

**DOI:** 10.1371/journal.pone.0187110

**Published:** 2017-11-06

**Authors:** Maddalena A. Wu, Francesco Casella, Francesca Perego, Chiara Suffritti, Nada Afifi Afifi, Eleonora Tobaldini, Andrea Zanichelli, Chiara Cogliati, Nicola Montano, Marco Cicardi

**Affiliations:** 1 Department of Biomedical and Clinical Sciences, University of Milan, Milan, Italy; 2 Department of Clinical Sciences and Community Health, IRCCS Ca’ Granda Foundation, Ospedale Maggiore Policlinico, University of Milan, Milan, Italy; Max Delbruck Centrum fur Molekulare Medizin Berlin Buch, GERMANY

## Abstract

**Background:**

Attacks of Hereditary Angioedema due to C1-inhibitor deficiency (C1-INH-HAE)are often triggered by stressful events/hormonal changes.

**Objective:**

Our study evaluates the relationship between autonomic nervous system (ANS) and contact/complement system activation.

**Methods:**

Twenty-three HAE patients (6 males, mean age 47.5±11.4 years) during remission and 24 healthy controls (8 males, mean age 45.3±10.6 years) were studied. ECG, beat-by-beat blood pressure, respiratory activity were continuously recorded during rest (10’) and 75-degrees-head-up tilt (10’). C1-INH, C4, cleaved high molecular weight kininogen (cHK) were assessed; in 16 patients and 11 controls plasma catecholamines were also evaluated. Spectral analysis of heart rate variability allowed extraction of low-(LF) and high-(HF) frequency components, markers of sympathetic and vagal modulation respectively.

**Results:**

HAE patients showed higher mean systolic arterial pressure (SAP) than controls during both rest and tilt. Tilt induced a significant increase in SAP and its variability only in controls. Although sympathetic modulation (LFnu) increased significantly with tilt in both groups, LF/HF ratio, index of sympathovagal balance, increased significantly only in controls.

At rest HAE patients showed higher noradrenaline values (301.4±132.9 pg/ml vs 210.5±89.6pg/ml, p = 0.05). Moreover, in patients tilt was associated with a significant increase in cHK, marker of contact system activation (49.5 ± 7.5% after T vs 47.1 ± 7.8% at R, p = 0.01).

**Conclusions:**

Our data are consistent with altered ANS modulation in HAE patients, i.e. increased sympathetic activation at rest and blunted response to orthostatic challenge. Tilt test-induced increased HK cleavage suggests a link between stress and bradykinin production.

## Introduction

Angioedema, is a localized self-limiting edema associated with different mechanisms. It can arise with wheals in a setting of allergic reactions or of chronic spontaneous urticarial as a result of mast cell degranulation. Angioedema without wheals can still be mediated by histamine, but can also be independent of mast cells and in this event it stands as a separate entity and can be inherited or acquired. In 2014 a comprehensive classification of “angioedema without wheals” was released, identifying three types of hereditary angioedema (genetic C1-INH deficiency, normal C1-INH with Factor XII mutations and unknown origin) and four types of acquired angioedema (C1-INH deficiency, related to ACE inhibitors intake, idiopathic histaminergic and idiopathic non-histaminergic) [1].

The best-characterized form is hereditary angioedema (HAE) due to C1 inhibitor deficiency (C1-INH-HAE). Symptoms occur episodically upon release of bradykinin resulting from hyperactivation of the contact system lacking its main control protein C1-INH [2]. They can affect cutaneous districts, as well as the gastrointestinal and upper airway mucosa. Angioedema can be insignificant, but most of the time it causes disability with personal and social consequences such as disfiguration and severe abdominal pain. When it localizes to the larynx, it can be lethal and more than 25% of affected subjects suffocate if appropriate treatments are not immediately available [3]. Disease burden depends on how often disabling events occur and on how they impact the subject’s life [4–6].

The frequency and severity of angioedema recurrences differ from patient to patient and in the same patient throughout life. C1-INH plasma levels do not satisfactorily account for the variability in clinical phenotypes [7]. In angioedema recurrences, the release of bradykinin occurs locally and is facilitated by trauma and psychological stress [8,9]. While it sounds logical to speculate that endothelial cells injured by trauma could become local activators of the contact system, pathways linking psychological stress to increased endothelial permeability are not obvious [10]. Stress may affect organ functions through the autonomic nervous system (ANS) and the hypothalamic-pituitary-adrenal axis [11]. These pathways can modify blood flow and endothelial barrier, thus increasing vascular permeability [12]. The ANS orchestrates these effects with a net influence on permeability. Sympathetic nervous system (SNS) inhibition with clonidine, an α_2_ agonist, reduces microvascular permeability in endotoxemic animals suggesting that antagonizing SNS might prove beneficial in stabilizing capillary leakage during inflammation [13]. On the other side the vagus nerve has a protective role in models of inflammation such as ischemia–reperfusion injury [14], viral myocarditis and postoperative ileus; it also decreases plasma TNFα concentrations after LPS administration [15–17]. Hence, sympathetic/parasympathetic balance can influence the vascular response to inflammatory stimuli. Investigating the ANS could provide new insights into the understanding of angioedema, a phenomenon due to an apparently incongruous action of inflammatory peptides.

Assessing ANS activity in humans is notoriously difficult. Experimental evidence supports the notion that the respiratory rhythm of heart period variability, defined as the high frequency (HF) spectral component, is a marker of vagal modulation. The rhythm defined as low frequency (LF), present in heart rate and systolic arterial pressure (SAP) variabilities, is a marker of sympathetic modulation [18,19]. In physiological conditions, a reciprocal relation exists between the relative amplitude of these two rhythms that is similar to that characterizing the sympathovagal balance [19]. Heart rate variability (HRV), described as the sum of elementary oscillatory components in the frequency domain, unveils both cardiac sympathetic and parasympathetic modulations. It provides a non-invasive, *in vivo*, physiological approach to identify the rhythmical components that allow evaluation of autonomic modulation. Several observational studies have found inverse relationships between vagal HRV parameters and markers of inflammation [20–23].

Here we analyzed ANS modulation together with cleaved high molecular weight kininogen, a biochemical marker of disease severity in patients with C1-INH-HAE, at steady state and in a controlled condition of stress induced by the orthostatic challenge (tilt testing) [24,25].

## Materials and methods

### Study overview and population

The study was designed by the investigators and all patients and controls provided written informed consent. The protocol adheres to the principles of the Declaration of Helsinki.

The protocol was approved by L. Sacco Hospital Ethics Committee (Protocol 2015/ST/253).

The study cohort consisted of C1-INH-HAE patients followed at the Department of Biomedical and Clinical Sciences, University of Milan and normal subjects recruited among people working or studying in this Department. All the clinical, laboratory, genetic or statistical information regarding the human subjects are stored at the Department of Biomedical and Clinical Sciences, Division of Internal Medicine, University of Milan, L.Sacco Hospital.

23 C1-INH-HAE patients 20 of whom are type 1 and 3 are type 2 (6 males, mean age 47.5±11.4 years, mean BMI 23.4 ± 4.8 kg/m^2^) during remission and 24 healthy volunteers (8 males, mean age 45.3±10.6 years, mean BMI 24.5 ±3.2 kg/m^2^) were studied. Patients and controls under 18 years of age, patients with ongoing or recent (≤ 8 days) acute attack, patients receiving acute HAE treatment within the last 8 days, patients and controls hospitalized for any ongoing clinical condition, patients and controls with relevant comorbidities and pregnant women were excluded from the study. Chronic ongoing medications were maintained, except for those (as beta blockers) with proved direct effect on ANS.

The day of the test, the subjects enrolled in the study avoided smoking, drinking coffee or alcohol-containing beverages and any stressful activity.

Specific HAE drugs were available in site, if needed to treat acute attacks.

### Tilt test and recordings

The tilt test was used as a tool to induce a “controlled stress” via the orthostatic challenge [26].

The subjects were supported on the tilt table by two belts at the thigh and waist, respectively, and with both feet touching the footrest of the tilt table.

A three lead-ECG, beat-by-beat plethysmograph arterial blood pressure (Finapress Medical Systems) and respiratory movements (via a thoracic piezoelectric belt) were recorded.

The signals were recorded at a sample frequency of 1000 Hz. Traces were recorded continuously for 10 minutes in clinostastism (rest) and for 10 minutes in orthostatism using a head-up tilt table with a 75° inclination (tilt). Blood pressure was also measured manually by the investigators using a standard sphygmomanometer at the beginning and at the end of the recording sessions to check the correspondence with the beat-to-beat measurements.

During the entire sessions, the subjects breathed spontaneously but they were not allowed to talk at any time.

Heart Rate Variability analysis was performed on the time series to extract the rhythmic oscillations that characterize both HR and blood pressure (BP) time series. As to ECG analysis, after the detection of QRS complexes, the apex of the R wave was located using a parabolic interpolation. The heart periods were automatically calculated on a beat-to-beat basis as the time between two consecutive R peaks (RR interval). QRS detection was checked to avoid missed beats and incorrect detection of R waves. Occasional ectopic beats were identified and replaced with an interpolated RR interval. As to BP, the beat-to-beat time series of systolic BP were generated and the intervals between consecutive maximum values of the first derivative of the arterial pressure waveforms was taken as BP time series. The respiratory signal was sampled at the occurrence of the first QRS peak delimiting RR. The series were linearly detrended. Short stationary samples of 250±50 beats length were chosen in rest and tilt conditions.

Autoregressive spectral analysis allowed us to identify the main oscillatory patterns embedded in the signal: low-frequency (LF, ranging from 0.04 to 0.15 Hz) oscillations, markers of sympathetic modulation; high-frequency (HF, ranging from 0.15 to 0.4 Hz) component, marker of parasympathetic modulation, synchronous with respiration.

These oscillations are characterized by specific frequency band and amplitude. Both LF and HF can be expressed in absolute values of power (ms^2^ and mmHg^2^ for RR interval and BP respectively) and in normalized units (nu), which represent the relative value of each spectral component in proportion to the total power. Normalized units can be calculated as follows: LF nu = [LF absolute units / (total power–VLF power)] and the HF nu = [HF absolute units/(total power-VLF)]. The sympathovagal balance is expressed by the calculation of the ratio between LF and HF power (LF/HF): the lower the LF/HF, the lower the sympathetic modulation and viceversa. We calculated the variation between rest and tilt for each spectral variable within each population (HAE patients and controls). Finally we compared controls to HAE patients.

### Blood sampling

Blood samples were collected using sodium citrate 3.2% as anticoagulant. The samples were centrifuged at 2000 g for 20 min at room temperature; the plasma was divided into aliquots, and stored at -80°C until tested.

For cleaved HK, blood, obtained with clean venipuncture and minimal stasis, was drawn into vacutainer tubes containing specific anti protease mixture [27]. Samples were centrifuged within one hour for 20 minutes at 2000 g and 20°C. The plasma was removed, divided into aliquots and stored at –80°C until tested. Samples were thawed quickly at 37°C immediately before the assays.

### Laboratory methods

C1-INH, C1q, and C4 antigens were measured by radial immunodiffusion (RID) (NOR-Partigen, Siemens Healthcare Diagnostics, Munich, Germany).

C1-INH function was assayed for capacity of plasma to inhibit the esterase activity of exogenous C1s measured on a specific chromogenic substrate by a commercial kit (Technoclone GmbH, Wien, Austria).

The cleavage of HK was assessed by means of sodium dodecyl sulfate-polyacrylamide gel electrophoresis (SDS-PAGE) and immunoblotting analysis [28,29]. Samples were loaded on a 9% SDS-polyacrylamide gel. After electrophoretic separation, proteins were transferred from the gel to a polyvinylidenedifluoride membrane using Bio-rad Trans-Blot^®^ Turbo^™^ Transfer System (Bio-Rad Laboratories, Hercules, USA). HK was identified by goat polyclonal anti-HK light chain (Nordic, Tilburg, the Netherlands) and visualized with a biotinylated rabbit antigoat antibody (Sigma Aldrich Co, St Louis, USA). The density of the bands was measured using a Bio-Rad GS800 densitometer and the amount of cleaved HK was expressed as a percentage of total HK.

### Statistical analysis

Data were elaborated with SPSS Statistics 23.0 calculating mean ± standard deviation.

Paired t-test was be used to assess significance among the different conditions (baseline and orthostatic challenge) within each population (controls and HAE patients). Unpaired t-test was used to compare the two populations (HAE patients and healthy volunteers) both at baseline and during 75°head-up tilt.

p< 0.05 was considered statistically significant.

## Results

Given that spectral methodology can be applied only to relatively stationary signals, time series of poor quality had to be discarded, whereas data from 20 C1-INH-HAE patients and 19 healthy volunteers were suitable for HRV analysis.

Table 1 shows the characteristics of patients and controls included in the study. The characteristics of patients whose signals were suitable for HRV analysis are specified too.

**Table 1:** Characteristics of patients and controls enrolled in the study.

	Patient Characteristics		Control Characteristics	
	Whole cohort (23 patients)	Cohort suitable for HRV analysis (20 patients)	Whole cohort (24 controls)	Cohort suitable for HRV analysis (19 controls)
**Gender**				
Female	17	14	16	14
Male	6	6	8	5
**Age**				
Mean age in the whole cohort	47.5±11.4	49±11.4	45.3±10.6	47±10.6
Mean age in males	38.3±9.0	38.3±9.0	43.0±11.1	47.2±12.1
Mean age in females	50.7±10.5	53.6±9.2	47.4±9.9	47.5±10.6
**BMI**	23.4±4.2	23.7±4.4	23.8±3.1	23.8±3.3
**Angioedema type**				
Type I	20	17	N/A	N/A
Type II	3	3	N/A	N/A
**Prophylaxis**				
None	6	5	N/A	N/A
Lanadelumab	2	1	N/A	N/A
Cinryze	1	1	N/A	N/A
Recombinant C1- inhibitor	1	1	N/A	N/A
Androgens	13	12	N/A	N/A

HR, RR interval (RRi) and respiratory rate (RespR) in C1-INH-HAE patients and controls were not significantly different during both rest and tilt. In both groups HR response to orthostatic challenge was preserved, with a significant increase of HR after tilt and no changes in RespR [Table 2].

**Table 2:** HRV parameters of 20 C1-INH-HAE patients and 19 controls (rest and tilt).

		Mean±SD	Patients vs controls at R	Mean±SD	Patients vs controls after T	R vs T in patients and in controls
**Heart rate (bpm)**	C1-INH-HAE-patients	71.2±12.6	N.S.	81.6±10.8	N.S.	<0.001
	Controls	72.7±13.5		83.3±11.9		<0.001
**RRi (ms)**	C1-INH-HAE-patients	872.3±147.9	N.S.	795.5±108.7	N.S.	<0.05
	Controls	868.6±138.6		769.7±204.8		<0.001
**LF nu**	C1-INH-HAE-patients	57.7±24.9	N.S.	69.7±26.1	N.S.	<0.05
	Controls	51.5±21.2		78.0±20.7		<0.001
**HF nu**	C1-INH-HAE-patients	26.3±19.7	N.S.	23.0±18.4	0.05	N.S.
	Controls	31.5±17.4		13.2±12.1		<0.001
**LF/HF**	C1-INH-HAE-patients	5.3±8.3	N.S.	12.8±21.6	N.S.	N.S.
	Controls	2.7±2.9		16.5±18.7		<0.01
**SAP (mmHg)**	C1-INH-HAE-patients	134.0±19.0	<0.001	141.4±28.8	0.01	N.S.
	Controls	114.9±9.8		121.7±17.3		<0.05
**RespR (breaths/min)**	C1-INH-HAE-patients	17.8±2.9	N.S.	18.2±5.8	N.S.	N.S.
	Controls	18.3±2.7		18.6±4.2		N.S.
**HF (Hz)**	C1-INH-HAE-patients	0.29±0.04	N.S.	0.30±0.06	N.S.	N.S.
	Controls	0.30±0.04		0.30±0.08		N.S.

SAP was significantly higher in C1-INH-HAE patients than in controls both at R and during T (Table 2). Tilt test induced an increase in mean SAP, which was statistically significant in the control group, but not in the HAE patients’ group.

During T, HF nu was higher in C1-INH-HAE patients than in controls with lower variation of HF (23.0±18.4 vs 13.2±12.1, p = 0.05 and -3.3±16.7 vs -18.4±17.2, p<0.01 respectively) (Table 2).

LFnu increased significantly after orthostatic challenge in both groups (Table 2), but only in healthy subjects there was a significant increase of LF/HF ratio, an index of sympathovagal balance (Table 2).

As expected, plasma C1-INH antigen and function as well as C4 antigen were markedly lower in C1-INH-HAE patients than in controls. Both proteins showed a tendency to increase after tilt (R/T values: C1-INH Ag 24.0 ± 34.5%/27.6 ± 33.4 in patients and 100.7 ± 10.6%/102.2 ± 7.8% in controls; C1-INH Fx 19.2 ± 13.1%/24.1 ±13.8 in patients and 86.5 ± 27.5%/94.7 ± 24.3% in controls; C4 29.6 ± 27.3%/30.3 ± 25.3% in patients and 102.1 ± 23.0/105.0 ± 21.0% in controls). The increments were statistically significant for C1-INH antigen and function in patients, and only for C1-INH function in controls (p = 0.04) (Table 3).

**Table 3:** Biochemical parameters of 23 C1-INH-HAE patients and 24 controls (rest and tilt).

Parameter	Subjects	Rest	p	Tilt	p	p
		Mean ± SD	Patients vs controls at R	Mean ± SD	Patients vs controls after T	R vs T in patients and in controls
**C1-INH Fx (%)**	C1-INH-HAE-patients	19.2 ± 13.1	<0.001	24.1 ±13.8	<0.001	<0.01
	Controls	86.5 ± 27.5		94.7 ± 24.3		<0.05
**C1-INH Ag (%)**	C1-INH-HAE-patients	24.0 ± 34.5	<0.001	27.6 ± 33.4	<0.001	<0.05
	Controls	100.7 ± 10.6		102.2 ± 7.8		N.S.
**C1q (%)**	C1-INH-HAE-patients	100.5 ± 6.1	N.S.	100.2 ± 7.0	N.S.	N.S.
	Controls	102.9 ± 5.0		103.3± 5.0		N.S.
**C4 (%)**	C1-INH-HAE-patients	29.6 ± 27.3	<0.001	30.3 ± 25.3	<0.001	N.S.
	Controls	102.1 ± 23.0		105.0 ± 21.0		N.S.
**cHK (%)**	C1-INH-HAE-patients	47.1 ± 7.8	<0.001	49.5 ± 7.5	<0.001	0.01
	Controls	38.7 ± 7.2		40.0 ± 6.1		N.S.

Noradrenaline was higher in patients at R (p = 0.05) and increased in both groups after tilt test (R/T values: 301.4±132.9pg/ml/467.4 ± 193.6 pg/ml, p<0.001 in C1-INH-HAE patients; 210.5 ± 89.6/407.4 ± 128.5 pg/ml in the controls, p< 0.001).

Cleaved kininogen (cHK) is increased in C1-INH-HAE patients compared to controls [24] and tilt test induced a significant increase in cHK only in C1-INH-HAE patients (Table 3).

## Discussion

A major finding of our study is that patients with C1-INH-HAE, and no concomitant co-morbidities, have an increased sympathetic modulation at rest, associated with a blunted response to a sympatho-excitatory stimulus such as head-up tilt. Moreover, tilt test in these patients was associated with a significant increase in HK cleavage, confirming a correlation between stress and bradykinin production.

In the first description of families with hereditary angioedema, Quincke termed the edema as angioneurotic. Later, HAE was interpreted as “a familial paroxysmal dysfunction of the autonomic nervous system, often precipitated by emotional stress” [30]. In the present study, we assessed the autonomic nervous system (ANS) through the analysis of heart rate variability (HRV). Applying this methodology, we found that C1-INH-HAE patients present an altered sympathovagal balance with signs of sympathetic excitation at baseline and blunted response to sympathetic stimulation provoked by the orthostatic challenge of the tilt test, i.e. of a standardized form of physical stress. These findings point to C1-INH-HAE as a disease with underlying autonomic dysfunction and confirm the assumption of Heiner et al. In parallel to ANS parameters, we measured plasma levels of the complement components C1-INH and C4. We also measured the cleaved high molecular weight kininogen (cHK), a breakdown product created when plasma kallikrein releases bradykinin from HK [31]. During angioedema attacks, levels of cHK increase in C1-INH-HAE patients’ plasma [29]. We previously showed that plasma levels of this byproduct have a positive correlation with attack frequency [24]. Therefore, cHK stands as biomarker of disease severity in patients with C1-INH-HAE. While the production of complement proteins induced by tilt were similar in patients and controls, cHK increased significantly only in patients, suggesting that such an activation occurs at a higher degree due to the deficiency of the physiologic regulator. Somehow, the sympathetic stimulus used in our study appears to facilitate one of the biochemical changes that characterizes occurrence of angioedema attacks [24].

Angioedema of any origin has a high prevalence in the general population [32]. A large number of angioedema is allergic, associated with exposure to specific agents. Aside from that, there are subjects with angioedema recurrences not related to specific causative factors. They carry conditions, only seldom recognized, that lower the threshold level of angioedema appearance. Such conditions can be in the blood stream, as are most vasoactive mediators; inside the endothelial cells, as pathways that modify intercellular junctions; or around the vessels as nerves that change blood flow. Individual angioedema threshold level is the net result of interactions between all vasoactive factors. Specific mutations in C1-INH (*SERPING1*) and *Factor12* genes alter the contact system and lower the angioedema threshold to clearly pathological levels [33]. Exposure to angiotensin converting enzyme inhibitors (ACEi) reduces catabolism of bradykinin and increases its plasma levels [34]. Yet, less than 1% of the general population develops recurrent angioedema when exposed to ACEi [35]. The side effect is well explained in subjects with decreased activity of serum amino-peptidase P or of dipeptidyl peptidase IV (DPPIV) that reduce bradykinin catabolism [36,37]. Many times, ACEi related angioedema remains unexplained and 20% of subjects who develop recurrent angioedema on ACEi continue to have angioedema after drug withdrawal [38]. We assume that in these subjects undisclosed variants increasing the levels of or the sensitivity to bradykinin operate to lower the angioedema threshold level. Mechanisms that facilitate endothelial permeability are under investigation. Vasoactive substances such as bradykinin, histamine, vascular endothelial grow factors (VEGFs) etc., act on endothelial cells through specific receptors that signal the intracellular nitric oxide pathway ending on retraction of tight and adherens junctions and fluid extravasation [39]. Recently, specific attention was paid to the possibility that an upregulation of different vasoactive substances may influence the risk of angioedema in C1-INH-HAE patients. E selectin, VGEF A and C and angiopoietin 2 are increased in these patients during intercritical periods and seem to predispose them to attacks [40–42]. Studying plasma markers of endothelial cells during angioedema attacks, Kajdacsi and colleagues found an activated phenotype suggesting that these cells actively participate in angioedema formation [43]. Our present data point to ANS as an additional player. The system is organized to sense through afferent fibers and to provide an adaptive response through the efferent pathways. We cannot distinguish whether the dysautonomia detected in C1-INH-HAE patients is primarily present, as hypothesized in 1957 [30], or if it is a consequence of the disease state. Alteration of sympathovagal balance at baseline and reduced response to an excitatory stimulus, as in our patients, are features of autonomic disturbances that characterize several diseases involving vascular endothelium (e.g. essential arterial hypertension, acute and chronic phases of myocardial ischemia, congestive heart failure, ulcerative colitis) [19,44–47]. This evidence and the unlikelihood of genetic dysautonomia segregating along with a genetic plasma protein deficiency, suggest that the sympathetic activation is secondary to changes induced by the contact system on endothelial cells. Subjects genetically deficient in C1-INH present levels of bradykinin higher than normal subjects [48]. A mouse model of C1-INH deficiency showed an ongoing bradykinin-dependent increase in vascular permeability [49]. Thus, different lines of evidence indicate that in C1-INH-HAE patients the vascular endothelium is also abnormally stimulated in resting conditions.

The last point is whether ANS and/or its abnormal profile may in turn affect susceptibility to angioedema development. ATP is released as a co-transmitter with noradrenaline from sympathetic perivascular nerves to cause constriction. On the other side, ATP released from endothelial cells, by the shear stress produced by blood flow and by hypoxia, produces endothelium-derived relaxing factor(s) [50]. Thus, endothelial cells mediate vasodilation to counterbalance the vasocontractile effects of ATP and noradrenaline released from perivascular sympathetic nerves. We postulate that increased efferent sympathetic nerve activity enables the release of the inflammatory peptide bradykinin. While it is relatively well accepted that the ANS protects from excessive inflammation [51], its role as trigger of an inflammatory process is still controversial. In an animal model, Jänig and Green found that bradykinin-induced plasma extravasation is largely dependent on the sympathetic innervation, but not on activity in the sympathetic neurons and not on the release of norepinephrine. It involves the hypothalamic-pituitary-adrenal (HPA) system, the sympatho-adrenal (SA) system, the nociceptive-neuroendocrine reflex (under control of vagal afferents) and the forebrain [52]. A connection between ANS and activation of the complement and contact systems can also be postulated. Endothelial cell heat shock protein 90 (Hsp90), identified as initiator of the contact system and the bradykinin-forming cascade [53,54], belongs to the so-called “stress proteins” and it is highly expressed or secreted during ‘‘stressful” conditions (such as hypoxia, infection, inflammation, exercise, exposure of the cells to toxins) or endothelial cell activation by cytokines [55–59]. In our study, a sympathetic stimulus causes small increase in a plasma marker of contact system activation and this increase reaches statistical significance in C1-INH-HAE patients. All together, these studies suggest that sympathetic activation (as during tilt test orthostatic challenge) can lead to increased HK cleavage, BK generation and eventually to angioedema attacks.

The aim of our study was to consider a random population of HAE patients in absence of acute or concurrent events of any type. This is the reason why, for instance, ongoing prophylaxis was not listed in the exclusion criteria. Among the 20 patients whose signals were suitable for HRV analysis 5 patients were not receiving any prophylaxis, 1 had been enrolled in the DX-2930 study with lanadelumab (human monoclonal antibody that targets plasma kallikrein), 1 was using Cinryze, 1 was under recombinant C1- inhibitor prophylaxis and 12 were taking androgens. However, we think that selection bias would have been a concern if we had enrolled only patients without prophylaxis because there would have been the risk of analyzing data only from patients with a less severe degree of disease (thus not requiring prophylaxis). Even though androgens might cause an increase of blood pressure in the long term, there is no evidence that they can act on autonomic modulation per se, namely the response to a controlled stress as tilt test. Moreover, even though It might be possible that androgen treatment modify ANS response, we decided not to exclude those subjects taking medication as part of their life since we were interested in investigating the ANS modulation in HAE patients and not in genetic C1-INH deficiency per se. Needless to say, nearly one third of the patients take prophylaxis and it would have been unethical to stop it for the study.

Although we are aware of limitations of the study related to the small sample size and of the carefulness needed not to emphasize too much the importance attributed to the statistical significance as expressed by the P value, we believe that our results are valuable starting points for further analysis, also considered that hereditary angioedema due to C1 inhibitor deficiency is a rare disease and that the study was demanding both for the patients and the investigators. So far to the best of our knowledge, besides case reports regarding the association of “stressful conditions “in general (as psychological upsets) to angioedema attacks, our study is among the first attempts to get a deeper insight into the interplay between these two “macro-systems”, Autonomic Nervous System and Contact-Complement System.

Further investigation is needed to expand existing knowledge and to confirm this hypothesis, but this preliminary evidence suggests that angioedema may be a useful model to reach better understanding of the relationship between autonomic nervous and immune inflammatory systems.

## Supporting information

S1 ANS Angioedema—De-identified data set. The raw data set from which results have been derived is available as de-identified data set included as a Supporting Information File.
(XLSX)

## Abbreviations

ACE: angiotensin converting enzyme
ANS: autonomic nervous system
BK: bradykinin
BMI: body mass index
BP: blood pressure
cHK: cleaved high molecular weight kininogen
C1-INH: C1 inhibitor
C1-INH Ag: antigenic C1 inhibitor
C1-INH Fx: functional C1-INH
HAE: hereditary angioedema
HF: high frequency
HK: high molecular weight kininogen
HPA: hypothalamic-pituitary-adrenal
HR: heart rate
HRV: heart rate variability
HSP: heat shock protein
LF: low frequency
LPS: lipopolysaccharide
nu: normalized units
RespR: respiratory rate
RRi: RR interval
SA: sympatho-adrenal
SAP: systolic arterial pressure
SNS: sympathetic nervous system
VEGF: vascular endothelial growth factor
